# Retraction: A comparative analysis of blended learning and traditional instruction: Effects on academic motivation and learning outcomes

**DOI:** 10.1371/journal.pone.0302484

**Published:** 2024-04-18

**Authors:** The PLOS ONE Editors

Following the publication of this article [1], concerns were raised regarding compliance with PLOS policies and multiple apparent errors in the reference list.

Specifically,

- Concerns were raised about potential undisclosed use of an artificial intelligence tool to generate text in the article due to inclusion of the phrase “regenerate response” and extensive reference list concerns. PLOS was unable to verify 18 of the 76 cited references, and 6 additional references appear to contain errors. The first and corresponding authors stated that the authors were responsible for the manuscript content and that the only AI tool used during manuscript preparation was Grammarly, to improve language. They provided replacement references but several of the replacements did not appear to support the corresponding statements in the article.
- The article [1] declared that ethics approval was obtained from Soochow University but did not mention approvals from Pakistan where the study was conducted. The first and corresponding authors stated that they sought permissions from Pakistani authorities before conducting the study. The ethics approval document and Pakistani approval documents provided to PLOS were dated in January 2023, after the recruitment start date listed in the S1 Checklist [1]. The recruitment dates in the S1 Checklist (June 2022 –April 2023) differ from those listed in the Materials and Methods section (January 2023 –July 2023). The first author asserted that the latter are the correct dates of recruitment but PLOS has been unable to obtain institutional input needed to clarify this issue.
- The *PLOS ONE* Editors have concerns about the article’s compliance with PLOS Authorship policy based on information that came to light during post-publication discussion with the authors.

The *PLOS ONE* Editors retract this article due to the above concerns which have not been fully resolved and which call into question the article’s reliability and compliance with the PLOS policies. The editors regret that these issues were not identified prior to publication.

MO and MY agreed with the retraction. RS, QH, and RM did not agree with the retraction. II either did not respond directly or could not be reached.
